# Effect of Motor Imagery on Corticomotor Excitability and Pain Status in Rheumatoid Arthritis Patients

**DOI:** 10.7759/cureus.42101

**Published:** 2023-07-18

**Authors:** Akanksha Arya, Abhishek Sinha, Raj Kumar Yadav, Srikumar Venkataraman, Uma Kumar, Renu Bhatia

**Affiliations:** 1 Department of Physiology, All India Institute of Medical Sciences, New Delhi, New Delhi, IND; 2 Department of Physiology, All India Institute of Medical Sciences, Guwahati, Guwahati, IND; 3 Department of Physical Medicine and Rehabilitation, All India Institute of Medical Sciences, New Delhi, New Delhi, IND; 4 Department of Rheumatology, All India Institute of Medical Sciences, New Delhi, New Delhi, IND

**Keywords:** tms, rheumatoid arthritis, rehabilitation, pain, motor imagery

## Abstract

Objectives: Rheumatoid arthritis (RA) has been defined by the American College of Rheumatology in 1987 as a chronic inflammatory disease characterised by joint swelling, joint tenderness, and destruction of synovial joints leading to severe disability and premature mortality. There is a paucity of literature assessing corticomotor excitability in RA patients. This study aimed to assess the effect of motor imagery on corticomotor excitability and pain status in RA patients. The specific objectives were to study the effect of motor imagery on corticomotor excitability and pain status in RA patients. We also wanted to compare the corticomotor excitability between RA patients with healthy controls. The correlation between the measures of corticomotor excitability and pain status in RA patients has also been done.

Methods: The study was designed as a pilot clinical trial with a case-control design. Forty participants were recruited for the study. Twenty RA patients were recruited from the Department of Rheumatology and Department of Physical Medicine and Rehabilitation (PMR), AIIMS, New Delhi, and 20 healthy controls. Testing was performed at the Pain Research & rTMS Lab, Department of Physiology, AIIMS, New Delhi. The study was approved by the Institute Ethics Committee, AIIMS New Delhi, and registered in the Clinical Trials Registry-India (CTRI). For the subjective assessment of pain, the visual analogue scale (VAS), Short-Form McGill Pain Questionnaire, WHO-Quality of Life Brief questionnaire (WHO-QOL-BREF), and Rheumatoid Arthritis Pain Scale were used. For the objective assessment of pain, hot and cold pain thresholds were assessed using thermo-tactile quantitative sensory testing (QST) using the method of limits and corticomotor excitability using a transcranial magnetic stimulation device. All participants were also asked to perform motor imagery tasks which consisted of a metronome-paced thumb opposition paradigm.

Results: The resting motor threshold (RMT) decreased significantly after motor imagery when compared to the mental calculation group. The amplitude of motor evoked potential (MEP) and QST parameter value was comparable in both the groups before and after motor imagery and mental calculation. RMT was found to be significantly higher whereas MEP values were found to be significantly lower in RA compared to controls.

Conclusion: We conclude that patients suffering from RA have decreased corticomotor excitability compared to controls. Motor imagery was effective in improving corticomotor excitability in these patients and can be used as rehabilitation in RA to relieve their pain.

## Introduction

Rheumatoid arthritis (RA) has been defined by the American College of Rheumatology in 1987 as a chronic inflammatory disease characterised by joint swelling, joint tenderness, and destruction of synovial joints leading to severe disability and premature mortality [1]. Symptomatic hand in RA patients is associated with disrupted movement and proprioception. These disruptions have primarily been attributed to the impairment in the periphery for example, in the joint capsule or the spinal cord. RA patients show increased pressure pain sensitivity and static muscle contraction reduces pressure pain among both persons with RA and control subjects with no major pain alike [2]. An RA patient reflects peripheral sensitisation within the dorsal horn or sensitisation of cortical regions [3]. In patients with RA, decreased pressure pain thresholds have been found both in structures overlying inflamed joints and in non-inflamed tissues, in conjunction with a significantly inverse correlation between the pressure pain thresholds and the intensity and duration of pain. Thermal thresholds in RA patients were significantly different from healthy controls [4]. The corticomotor excitability was studied in 13 women with fibromyalgia (FM) and five women with RA as well as 13 age-matched controls. Subjects completed one familiarisation and two experimental sessions. Motor evoked potential (MEP) of target muscles was measured following transcranial magnetic stimulation (TMS). This was the first study demonstrating that patients with FM, compared with controls, have significant impairment of motor evoked parameters linked with cortical excitatory and inhibitory mechanisms [5]. The relationship between chronic pain conditions like FM and osteoarthritis (OA) and the excitability of the motor cortex is studied in another study by TMS. Intracortical Inhibition was found to be reduced before exercise in Muscular Dystrophy and Fibromyalgia syndrome patients, whereas the same was seen in healthy subjects only during fatiguing muscle exercise [6]. Motor imagery (MI) is the ability to imagine performing a movement without executing it [7]. MI has been shown to share a similar mechanism of motor execution, i.e. premotor cortex and supplementary motor area seem to be involved in both tasks [7]. There is a bidirectional relationship between pain and motor cortex excitability [8]. Pain perception modifies TMS-induced cortical excitability in the motor cortex leading to alteration in corticomotor excitability in chronic pain conditions. It was hypothesised that MI, which is focused toward a specific hand, will significantly modify TMS-induced cortical excitability [9]. The effect of MI and pain perception in phantom limb patients was studied where 22 patients were allocated to one of three groups: those who viewed a reflected image of their intact foot in a mirror (mirror group), who viewed a covered mirror, and who were trained in mental visualisation task. Patients in the mirror group attempted to perform movements with the amputated limb while viewing the reflected image of the movement of their intact limb. Patients in the covered-mirror group attempted to perform movements with both their intact and amputated limbs when the mirror was covered by an opaque sheet while patients in the mental visualisation group closed their eyes and imagined performing movements with their amputated limb only. It was found that MI modulates pain in phantom limb pain [10]. Cortical plasticity was studied in the motor cortex following MI, using the paired associative stimulation (PAS) technique. A reversal of the PAS25 effect from long-term potentiation (LTP)-like plasticity to long-term depression (LTD)-like plasticity following physical and MI practice was seen in the study. While LTD-like plasticity (PAS10 protocol) increased after physical practice, it was occluded after MI practice. This study proved that MI did not just lead to cortical reorganisation but also strengthened the synaptic connectivity [11]. These findings suggest that neuronal plasticity is influenced by pain and that the mental imagery effects on pain depend on the state of central sensitisation. There is a paucity of literature that has explored the effect of MI on corticomotor excitability in RA patients. The aim of this study was to assess the effect of MI on corticomotor excitability and pain status in RA patients. The objectives were to study the effect of MI on corticomotor excitability and pain status in RA patients, to compare the measures of corticomotor excitability between RA patients with healthy control, and to correlate the measures of corticomotor excitability and pain status in RA patients.

## Materials and methods

The study was designed as a pilot clinical trial with a case-control design. Forty participants were recruited for the study; 20 RA patients were recruited from the Department of Rheumatology and Department of Physical Medicine and Rehabilitation (PMR), AIIMS, New Delhi, and 20 were healthy controls. Not a priori sample size calculation could be done due to a lack of comparable studies. Testing was performed at the Pain Research & rTMS Lab, Department of Physiology, AIIMS, New Delhi. The study was approved by the Institute Ethics committee, AIIMS New Delhi (Ref. No. IECPG-98/28.02.2019) and registered in the Clinical Trials Registry-India (CTRI) (REF/2019/05/025648 (B)). The inclusion criteria were RA patients with pain (more than 3 on the visual analogue scale [VAS]) for more than half the number of days in the past six weeks and having >6/10 score in the scoring system for RA given by the American College of Rheumatology. The right-handed patients were included. The participants of the control group were age- and gender-matched, pain-free healthy volunteers. The age group for both sets of subjects was 18-65 years . The exclusion criteria were patients with known contraindications to TMS including metal implants in the head (excluding dental fillings), known skull defects, facial tattoos, concussion within the last six months, history of epilepsy or seizures, a pacemaker or artificial heart valve, intracardiac lines, history of unexplained recurring headaches, and current pregnancy. Participants with a neurological condition, psychogenic/psychosomatic pain, a history of chronic pain other than RA, and cardiovascular and respiratory disorders were excluded. Participants who had a history of substance abuse were also excluded. For the subjective assessment of pain, VAS, Short-Form McGill Pain Questionnaire (MPQ-SF), WHO-Quality of Life Brief questionnaire (WHO-QOL-BREF), and Rheumatoid Arthritis Pain Scale were used. For the objective assessment of pain, hot and cold pain thresholds were assessed using thermo-tactile quantitative sensory testing (QST) using the method of limits and corticomotor excitability using a TMS device. All participants were also asked to perform an MI task, which consisted of a metronome-paced thumb opposition paradigm. 

Statistical analysis

Data were entered into MS Excel and analysed by SPSS 25.0 software for Windows (IBM Corp, Armonk, NY). The data obtained were screened for normality to decide the appropriate tests. The various statistical tests used for analysis are elaborated in the Results section.

## Results

**Figure 1:**
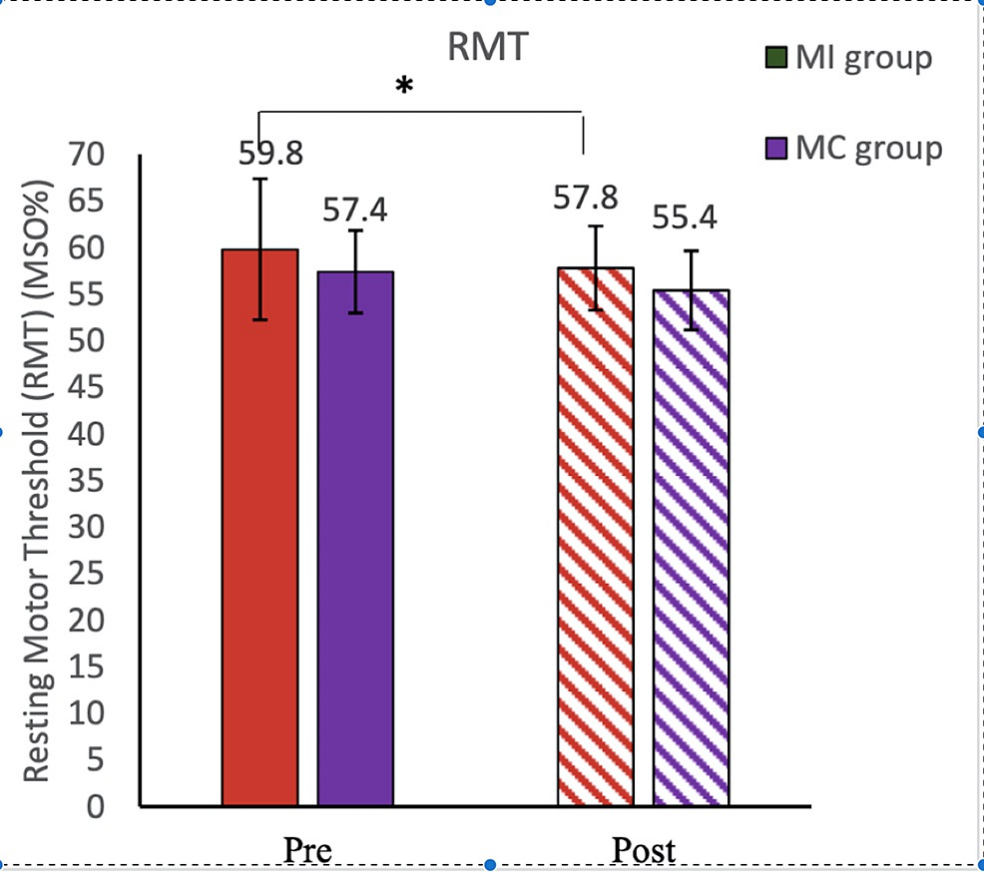
Resting motor threshold (motor imagery vs mental calculation group) This figure shows the RMT of the motor imagery group vs the mental calculation group. RMT decreased significantly in rheumatoid arthritis patients after motor imagery (P = 0.0245) and comparable after mental calculation (P = 0.0543). Data were checked for normality using the Shapiro-Wilk normality test. The comparison was done by ANOVA. Data have been expressed as mean ± SD. Asterisk sign represents the significant P-value. RMT, resting motor threshold; MI, motor imagery; MC, mental calculation; MSO, maximum stimulator output; ANOVA, analysis of variance.

**Figure 2:**
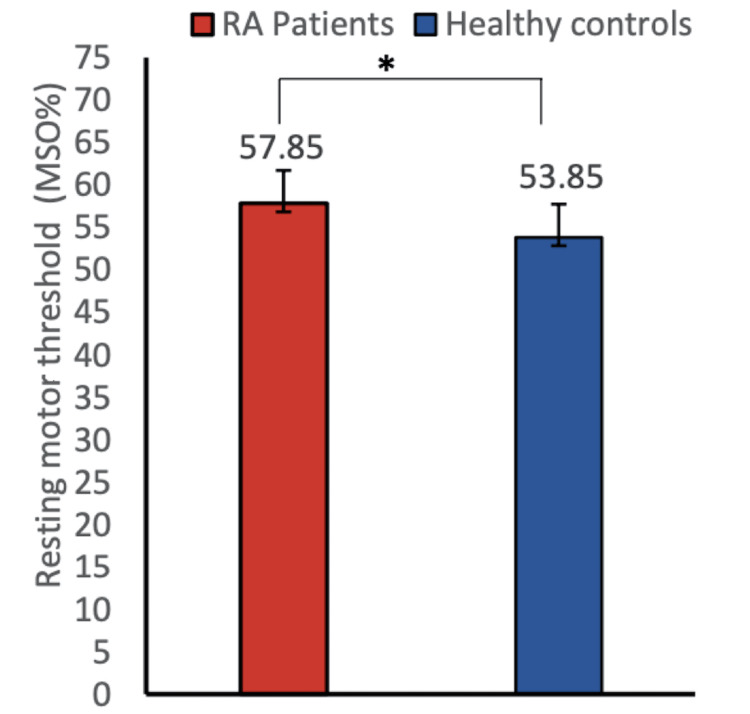
Resting motor threshold of RA patients and healthy controls This figure shows the comparison of RMT between healthy controls and rheumatoid arthritis patients. RMT was found to be significantly higher in rheumatoid arthritis patients as compared to healthy controls (mean ± SD 57.85 ± 6.64 vs. 53.85 ± 3.9, P = 0.0264). Data were checked for normality using the Shapiro-Wilk normality test. A two-group comparison was done by the standard t-test. Data have been expressed as mean ± SD. Asterisk sign represents the significant P-value. RA, rheumatoid arthritis; RMT, resting motor threshold.

**Figure 3:**
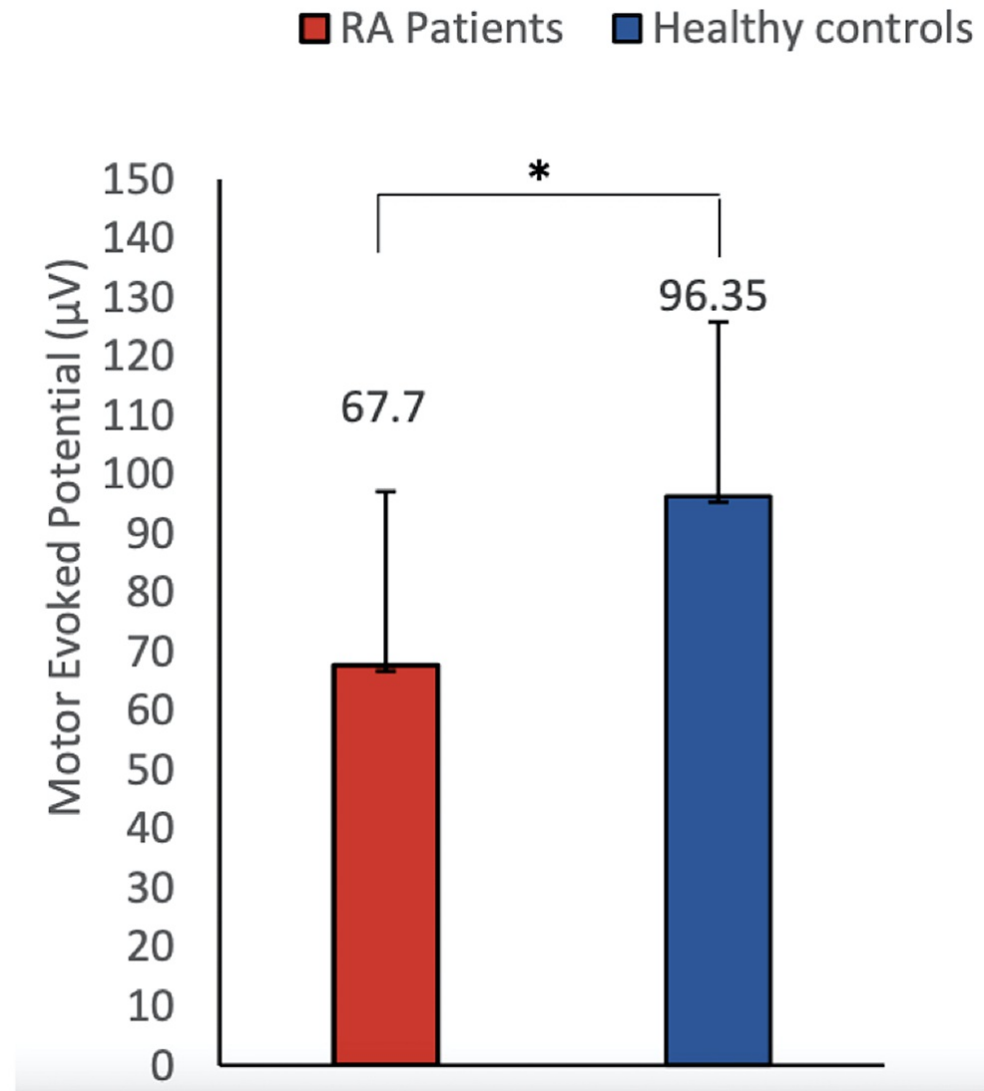
A comparison of MEP between RA patients and healthy controls This figure shows the MEP comparison between healthy controls and RA patients. MEP was found to be significantly lower in RA patients as compared to healthy controls (mean ± SD 67.7 ± 12.33 vs 96.35 ± 29.45; P = 0.0136). Data were checked for normality using the Shapiro-Wilk normality test. A two-group comparison was done by standard t-test. Asterisk sign represents the significant P-value. MEP, motor evoked potential; RA, rheumatoid arthritis.

**Figure 4:**
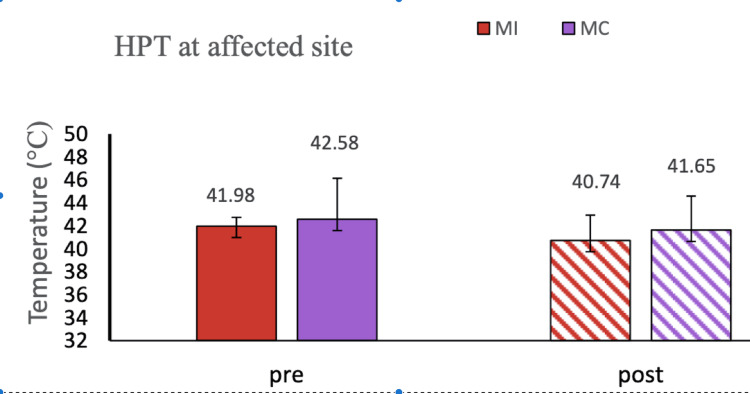
HPT at the affected site: MI vs MC group This figure shows the graphical representation of the QST parameter HPT at the affected site in the MI vs MC sham group. Data were checked for normality using the Shapiro-Wilk normality test. The comparison was done by ANOVA. Data have been expressed as mean ± SD. HPT, hot pain threshold; ANOVA, analysis of variance; MI, motor imagery; MC, mental calculation.

**Figure 5:**
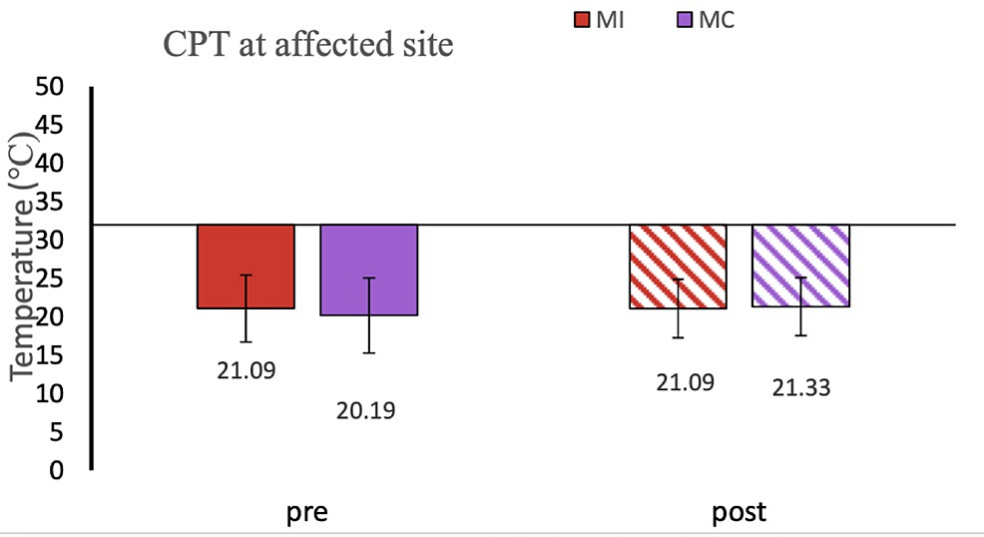
CPT at the affected site: MI vs MC group This figure shows the graphical representation of the QST parameter CPT at the affected site in the MI vs MC sham group. Data were checked for normality using the Shapiro-Wilk normality test. The comparison was done by ANOVA. Data have been expressed as mean ± SD. CPT, cold pain threshold; ANOVA, analysis of variance; MI, motor imagery; MC, mental calculation.

**Figure 6:**
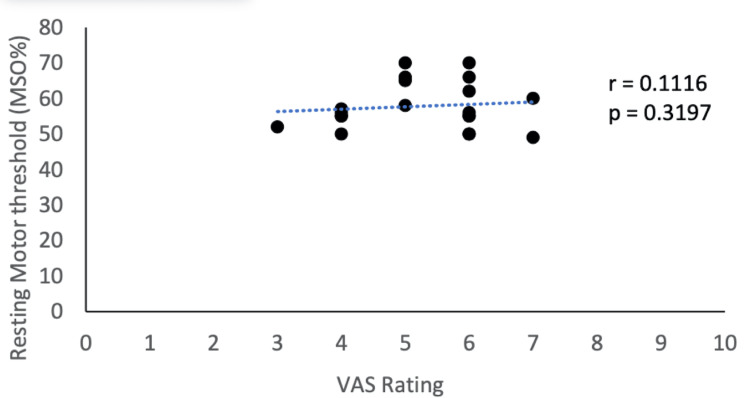
Correlation between corticomotor excitability and pain status This figure shows the correlation between corticomotor excitability (RMT) and pain status (VAS). Data were checked for normality using the Shapiro-Wilk normality test. A two-group correlation was done by Pearson's correlation coefficient test (r = 0.1116). RMT, resting motor threshold; VAS, visual analogue scale.

The demographic details of the participants are mentioned (Table 1.) RA patients reported moderate to severe pain on the numerical pain rating scale (VAS score = 5.5). In our study, we did not find any significant improvement in numerical scale rating (VAS score) after 5 minutes of MI as well as mental calculation task (Table 2). The WHO-QOL-BREF score was recorded in the MI and mental calculation groups and was found to be comparable at baseline (Table 3). The Rheumatoid Arthritis Pain Scale score (RAPS score) was also comparable before and after MI and mental calculation (Table 4). In the present study, high MPQ-SF scores (value = 26.35) were observed, suggesting the extreme nature of pain in RA patients (Table 5). We did not find any improvement in the corticomotor excitability parameters in the mental calculation group (Figure 1). In our study, we observed decreased amplitude of MEP whereas increased RMT in RA patients compared to healthy controls, suggesting lower corticomotor excitability in RA patients (Figures 2, 3). In our study, we used responses to thermal stimuli as an objective parameter for the assessment of pain status. We found the warm detection threshold (WDT) and cold detection threshold (CDT) to be comparable after MI in RA patients. The hot and cold pain thresholds (HPT, CPT) hot and cold pain tolerance thresholds (HPTT, CPTT) at baseline were higher in RA patients compared to healthy controls, which is similar to another study [2] (Figures 4, 5). The correlation between VAS scores and RMT in RA patients was not significant between the two parameters (Figure 6).

**Table 1:** Demographic characteristics of patients and healthy controls

Characteristics	Patients (n = 20)	Healthy controls (n = 20)
Age (years)	43.3 ± 7.36	39.45 ± 9.98
Female:male	18:2	18:2
Height (cm)	160 ± 10.9	164 ± 11.5
Weight (kg)	64 ± 12.4	58 ± 9.7
BMI (kg/m^2^)	24.6 ± 2	22.7 ± 1.4

**Table 2:** Representation of VAS of pain in rheumatoid arthritis patients before and after motor imagery and mental calculations. This table shows the comparison in resting motor threshold at baseline in the motor imagery and the mental calculation groups (P = 0.430). Data were checked for normality using the Shapiro-Wilk normality test. A two-group comparison was done by standard t-test. Data have been expressed as mean ± SD. VAS, visual analogue scale.

VAS	Motor imagery	Mental calculation
Pre-procedure	5.5 ± 0.97	5 ± 1.24
Post-procedure	5.1 ± 0.87	4.9 ± 1.1
P-value	0.103	0.342

**Table 3:** WHO-QOL-BREF questionnaire score at baseline in the motor imagery and mental calculation groups. This table shows the WHO-QOL-BREF questionnaire score at baseline in the motor imagery and mental calculation groups. Data were checked for normality using the Shapiro-Wilk normality test. The comparison was done by ANOVA. WHO-QOL-BREF, WHO-Quality of Life Brief; ANOVA, analysis of variance.

WHO-QOL-BREF score	Motor imagery	Mental calculation
Domain 1 (physical health)	56 ± 7.8	50 ± 12.8
Domain 2 (psychological)	50 ± 6.2	56 ± 8.6
Domain 3 (social relationship)	75 ± 12.7	56 ± 10.8
Domain 4 (environment)	69 ± 5.8	63 ± 6.8

**Table 4:** RAPS questionnaire score before and after motor imagery and mental calculation This table shows the RAPS questionnaire score before and after motor imagery and mental calculation. Data were checked for normality using the Shapiro-Wilk normality test. The comparison was done by ANOVA. RAPS, Rheumatic Arthritis Pain Scale; ANOVA, analysis of variance.

RAPS score	Motor imagery	Mental calculation
Pre-therapy	73.7 ± 17.1	74.5 ± 16.7
Post-therapy	73.7 ± 17.1	74.5 ± 16.7

**Table 5:** MSPF-SQ score of pain in rheumatoid arthritis patients before and after mental calculation This table shows the MPQ-SF score for rheumatoid arthritis patients for motor imagery vs mental calculation. The P-value was 0.663 for the motor imagery group and >0.99 for the mental calculation group. Data were checked for normality using the Shapiro-Wilk normality test. The comparison was done by ANOVA. MPQ-SF, McGill Pain Questionnaire short form; ANOVA, analysis of variance.

MPF-SQ score	Motor imagery	Mental calculation
Pre-procedure	26.35 ± 2.7	26.2 ± 1.6
Post-procedure	25.86 ± 2.2	26.0 ± 1.4
P-value	0.663	>0.99

RMT was found to be significantly higher whereas MEP values were found to be significantly lower in RA patients compared to healthy controls. RMT and subjective pain rating were not significantly correlated in RA patients. RMT decreased significantly after motor imagery when compared to that in the mental calculation group. The amplitude of MEP was comparable in both the groups before and after MI and mental calculation. Quantitative sensory testing (QST) parameters were comparable in both groups before and after MI and mental calculation.

## Discussion

RA is a chronic inflammatory disease characterised by the destruction of synovial joints leading to severe disability and premature mortality [1]. Corticomotor excitability can be assessed with the help of TMS of brain areas and assessing parameters like RMT and MEP. Earlier studies had established that MEP amplitude is an index of both presynaptic and postsynaptic activity and integrity, while RMT represents the excitability of a central core region of neurons [12-14]. Studies have shown that musculoskeletal pain can lead to an increase in RMT and a decrease in MEP amplitude [15,16]. MI is defined as the ability to imagine performing a movement without executing it [7], and action observation (AO) by subjects themselves has been shown to facilitate corticomotor excitability which is like that seen during actual movement of limb/muscle [17].

In our study, we observed decreased amplitude of MEP whereas increased RMT in RA patients compared to healthy controls suggesting lower corticomotor excitability in RA patients (Figures 2, 3). Our findings corroborate with the study of Salerno et al. [5] who found a lower amplitude of MEP in RA patients when compared to healthy controls. Another study reported that acute experimental pain exerts an inhibitory influence over the motor cortex, that can interfere with motor learning capacities [18]. There is a paucity of literature for the assessment of corticomotor excitability of RA patients; however, several studies that supported altered corticomotor excitability in other chronic pain conditions are present [5]. Lower MEP amplitude was observed in FM patients compared to healthy controls [5]. These studies support our findings and suggest reduced corticomotor excitability in patients with chronic pain conditions which is probably due to limited use or disuse of pain-associated affected limbs. Our results suggest a significant decrease in RMT after the MI task of thumb opposition (at the rate of 60 taps/minute for 5 minutes) in RA patients as well as in healthy controls, suggesting that MI can improve corticomotor excitability. Various studies using TMS demonstrated increased corticomotor excitability (RMT, MEP, CSP, cortical map extents) after the MI task [19]. Several studies using EEG have shown that corticomotor excitability changes are due to neural commonalities between MI and actual motor execution [11,20]. Functional magnetic resonance imaging (fMRI) studies have confirmed the activation of the same neural circuitry in MI as well as in motor execution [21]. The cortical areas that get activated during MI include the premotor and supplementary motor cortex area, parietal cortex area, cingulate gyrus, basal ganglia, cerebellum, and primary motor cortex [22-24]. Incidentally, these same areas are also activated during actual thumb movement. Our findings of decreased RMT after the MI task could be explained due to increased neuronal firing of cortex regions during the MI task [25].

We did not find any improvement in corticomotor excitability parameters in the mental calculation group (Figure 1). This could be attributed to the fact that during mental calculation, cortical areas activated are the left inferior parietal lobe, left precentral gyrus, left superior parietal lobe, left supramarginal gyrus, and left middle temporal gyrus, which are different from the cortical areas activated during motor execution and MI. Another study conducted on healthy subjects also concludes that neuronal plasticity is influenced by pain and that mental imagery can improve corticomotor excitability [11]. In another study, corticomotor excitability parameters were recorded in healthy controls at baseline, after induction of acute pain (hypertonic saline injection), and after 10 minutes of the MI task. They found that acute pain decreases corticomotor excitability (increased RMT, decreased MEP) but when participants performed the MI task, this effect on corticomotor excitability reverted back (decreased RMT, increased MEP) [26].

RA patients reported moderate to severe pain on the numerical pain rating scale (VAS score = 5.5) (Table 2). The WHO-QOL-BREF score was recorded in the MI and mental calculation groups and was found to be comparable at baseline (Table 3). Similar reports about pain intensity have been published [27]. In our study, we did not find any significant improvement in numerical scale rating (VAS score) after 5 minutes of MI as well as mental calculation task (Table 2). Improvement in numerical pain rating (VAS score) in RA patients after MI was seen in a similar study (7 minutes 3 times a day for 6 weeks). We suggest that the duration of the MI task is an important factor to achieve desirable results. The effect of mirror therapy on complex regional pain syndrome (CRPS) patients for six months was studied and there was a significant reduction in the pain rating of patients [28,29]. In our study, we failed to find improvement in pain score after 5 minutes of MI as an MI task of 5 minutes is insufficient to elicit the desirable results and previous studies have given increased duration of MI to achieve pain relief.

In the present study, a high MPQ-SF score (value = 26.35) was observed, suggesting the extreme nature of pain in RA patients (Table 5). There is a paucity of literature assessing the effect of MI on MPQ scores in RA patients. A similar study reported improvement in MPQ score after MI therapy for two weeks in CRPS patients [30]. The RAPS score was also comparable before and after MI and mental calculation (Table 4). The variable results of the studies could be due to the type of disability, pain, patient’s perception of pain, stress levels, patient’s expectation from treatment, and more importantly the MI protocol (whether it is mirror therapy, left and right discrimination, or explicit MI) that was administrated. In an attempt to understand the relationship between corticomotor excitability and pain scores, we studied the correlation between VAS scores and RMT in RA patients but there was no significant correlation between the two parameters (Figure 6).

In our study, we used responses to thermal stimuli as an objective parameter for the assessment of pain status. We found WDT and CDT to be comparable after MI in RA patients. The hot and cold pain thresholds (HPT, CPT, HPTT, and CPTT) at baseline were higher in RA patients compared to healthy controls which is similar to another study [2] (Figures 4, 5). After 5 minutes of MI task thermal thresholds did not change. A similar result was also reported by the healthy controls in another study [11].

Thus, we conclude that MI improves corticomotor excitability when used regularly for an extended duration. We propose that MI should be used as a rehabilitation intervention for RA patients.

Limitations and future directions

The present study was conducted with a limited number of patients, and we believe that a larger sample size would have provided better insights into the role of MI in pain relief in RA patients. The duration of MI may be increased for better pain relief. Also, the use of techniques such as fMRI could have elicited the actual brain areas activated during MI and mental calculation.

## Conclusions

We conclude that patients suffering from RA have decreased corticomotor excitability compared to age- and gender-matched healthy controls. MI was effective in improving corticomotor excitability in these patients. Hence, MI can be used as a rehabilitation intervention for RA, which can relieve RA patients from their pain and motor deficits.
